# *N*-Alkylhydantoins as New Organogelators and Their Ability to Create Thixotropic Mixed Molecular Organogels

**DOI:** 10.3390/gels8100638

**Published:** 2022-10-08

**Authors:** Yutaka Ohsedo

**Affiliations:** Division of Engineering, Faculty of Engineering, Nara Women’s University, Kitauoyahigashi-machi, Nara 630-8506, Japan; ohsedo@cc.nara-wu.ac.jp

**Keywords:** hydantoins, low-molecular-weight gelators, molecular gels, organogels, thixotropic behavior

## Abstract

The author reported molecular organogels using *N*-alkylhydantoins as new low-molecular-weight gelators for the first time, and thixotropic mixed molecular organogels using a set of *N*-alkylhydantoin gelators with different alkyl chain lengths. These homologous compounds with different alkyl chains are found to form macroscopic crystals or solution states in polar solvents, but form homogeneous organogels in non-polar solvents, such as *n*-octane and squalane. Although there is no significant increase in the minimum gelation concentration of the mixed molecular gels using squalane as a solvent, these mixed molecular organogels show improved mechanical properties, especially in their thixotropic behavior, which is not observed in the single *N*-alkylhydantoin gels. Furthermore, they exhibit reversible thixotropic behavior with quick recovery of the gel state in a minute by quantitatively measuring dynamic viscoelasticity measurements of rheometry of mixed molecular gels. Based on the morphological observations of the xerogels, the self-assembling fibers of the gelators become finer, indicating an increase in the density of the mesh structure inside the gel, which could explain its thixotropic behavior. These thixotropic mixed molecular gels may be applicable to ointment base materials, because they are gelled with squalane oil.

## 1. Introduction

As principal bioactive compounds, hydantoins, which contain an imidazolidine-2,4-dione known as glycolylurea, are important in medicinal chemistry [1,2]. The biochemical and biological activities of hydantoins on living things may originate from their cyclic urea and cyclic amide chemical structures. Owing to their variety of chemical structures, hydantoin derivatives have found many uses as an anticonvulsant, known as phenytoin [3]; as a pyrethroid pesticide, known as imiprothrin [4]; and as a preservative, known as MDM or DMDM hydantoin (1,2-dimethylol-5,6-dimethylhydantoine) for cosmetics [5]. In order to develop new hydantoins with high biological activities, several efforts were made to create an efficient synthetic route to obtain a variety of hydantoins [6]. In various derivatives of hydantoins, *N*-alkylhydantoins, which have been alkylated in one of the two N-positions of hydantoin, are used as additives in polymer materials for antimicrobial activity [7,8]. Initially, Baeyer first investigated the chemistry of hydantoins starting in 1861 [9]. Therefore, the chemistry of hydantoins consists of old and new positions in organic synthetic chemistry and medicinal chemistry.

On the other hand, in terms of materials chemistry, the chemical component units of *N*-alkylhydantoins, such as an alkyl chain, urea, and amide, may exhibit different inter-molecular interactions, resulting in anisotropic molecular self-assembly in its crystal formation, as shown in low-molecular-weight gelators (LMWGs) [10,11,12], which generally consist of the same types of chemical components as *N*-alkylhydantoins. Generally, LMWGs and a solvent produce molecular gels consisting of a network of self-assembled LMWG fibers with orders ranging from nanometers to micrometers and received significant attention as a new type of soft matter because of their unique multi-stimuli responses from the environment [13,14,15,16,17,18,19,20] and thixotropy, which undergo mechanically induced reversible gel-to-sol and sol-to-gel transitions [21,22,23,24,25,26,27,28]. Since thixotropy is an essential property of materials used as base materials for ointments, research on molecular gels has been actively conducted to obtain thixotropic molecular gels [29,30]. Therefore, creating new molecular gels with improved properties, especially in thixotropic behavior, is required to extend the usage of gels, such as in health care and medicinal fields [31,32]. However, although it is thought that *N*-alkylhydantoins function as LMWGs, such as alkylhydrazides [33], alkylureas [34], and alkylamides [35], there is no example of *N*-alkylhydantoins being used as LMWGs.

**Figure 1:**
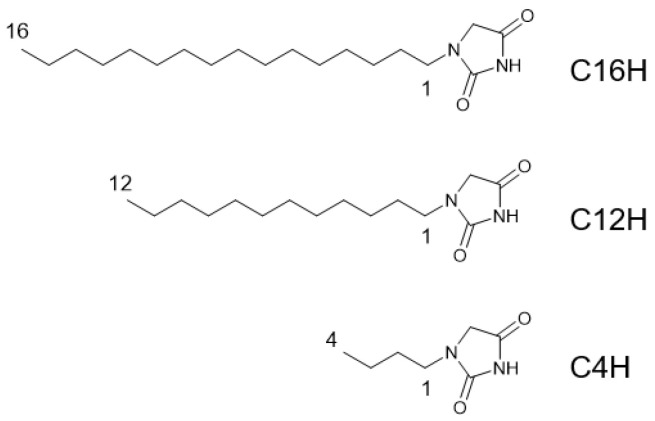
Chemical structures of *N*-alkylhydantoins in this study.

Here, the author reports a new creation of thixotropic molecular organogels using mixed *N*-alkylhydantoins with different alkyl chain lengths as LMWGs (Figure 1). Although creations of molecular gels with mixed gelators are reported [22,27,28,33,34,35,36,37,38,39,40,41,42,43,44,45,46,47], this is the first study to describe *N*-alkylhydantoins as LMWGs. This mixing of LMWGs with different alkyl chain lengths is shown as a new method for improving the mechanical properties of molecular gels, especially in thixotropy, which is probably due to the improved quality of the network of gels.

## 2. Results and Discussion

Commercially available *N*-alkylhydantoins (CnH), 1-*N*-butylhydantoin (C4H), 1-*N*-dodecylhydantoin (C12H), and 1-*N*-hexadecylhydantoin (C16H) were used, and their critical gel concentrations (CGC) for various organic solvents were examined (Table 1). From the results, *n*-octane, olive oil, and squalane are suitable solvents for CnH gels (all gels are opaque), and CnH with a longer alkyl chain seems better for forming gels in terms of CGC. These compounds are organogelators that function in non-polar solvents, such as olive oil and squalane. For C12H and C4H, with short alkyl chain lengths, except for hydrophobic solvents, the samples remain in solution after the gelation test, while for C16H, with longer alkyl chain lengths, crystals are formed, although no gel is formed, indicating a higher capacity for macroscopic crystal formation. In hydrophobic solvents, C16H has a lower CGC than C14H, which has a shorter alkyl chain length, suggesting that the longer the alkyl chain length, the better the ability to form gels. It is also discovered that longer alkyl chains are more favorable for gelation, probably because the magnitude of inter-molecular forces between alkyl group chains is more favorable for fiber formation [33]. Hereafter, the CnH/squalane system is the focus for studying the mixed gel system, owing to its better gel-forming properties. The mixed gel systems were performed only with mixed CnH in squalane (Table 2). Examinations of binary and tertiary systems of CnH/squalane systems prove that mixed gels in some compositions show improved CGC, such as the C16H/C4H 1/1 system (CGC 1.0 wt.%), despite C4H lacking gelation ability, and the C16H/C12H/C4H 1/1/1 system (CGC 1.0 wt.%). These mixed gels all show clear gels that retain their gel state for at least 6 months without crystallization. The results indicate that mixing CnHs for mixed molecular gels improves gel-forming properties.

**Figure 2:**
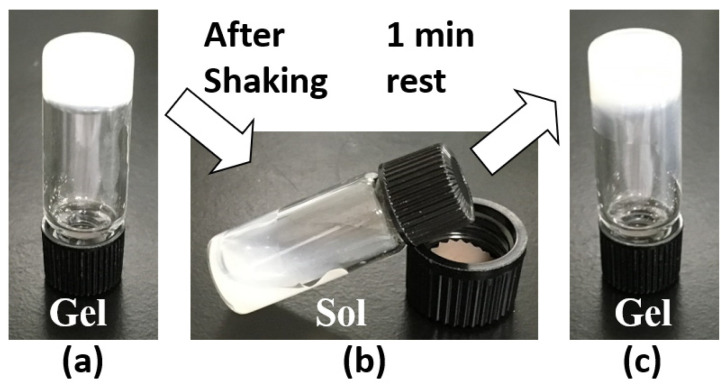
Thixotropic behavior of the organogel formed with C16H/C12H/C4H 1/1/1 (2 wt.%) squalane organogel: (**a**) organogel before shaking, (**b**) organosol after shaking, and (**c**) recovered organogel after 1 min rest of organosol.

In addition to the improvement in gel formation, the mixed molecular gel system demonstrates improved thixotropic behavior compared with the single system (Figure 2). As shown in Table 2, the tendency to recover from sol to gel is faster in mixed gels than in single gels. Although thixotropic behavior is not confirmed even at 3 wt.% in the single C16H and C14H squalane gels (no recovery to gel is observed after 1 day of standing), good thixotropic behavior is observed in some compositions in the two-component system and all compositions, except C16H/C12H/C4H 5/1/1, in the three-component system, with recovery from sol to gel occurring within 1 min. In the multicomponent system, there is no clear trend in the correlation between the mixing ratio and thixotropic behavior, and much remains unclear. However, the improvement in gel-forming ability and thixotropic behavior from a single-component system to a multicomponent mixture may be due to the difference in the network structure within the gel, which is discussed later.

**Figure 3:**
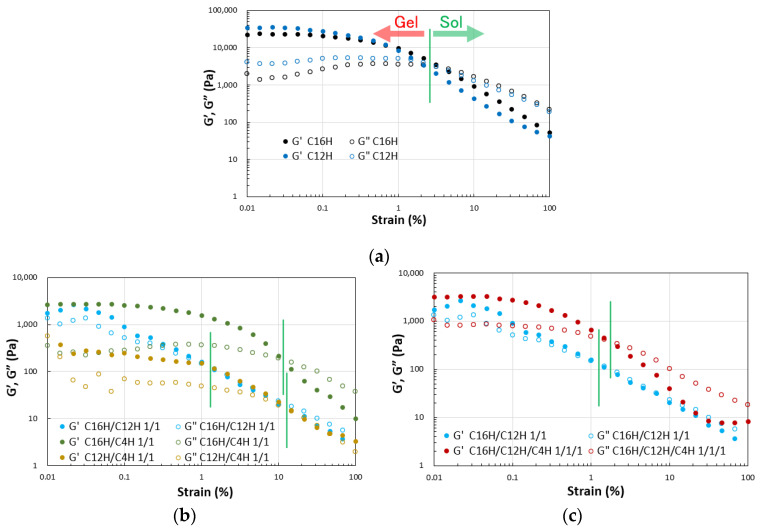
Dynamic rheological properties of the mixed molecular squalane organogels (3.0 wt.%) on strain sweep; (**a**) single gels, (**b**) binary gels, and (**c**) tertiary gels with C16H/C12H 1/1 gel as a reference.

**Figure 4:**
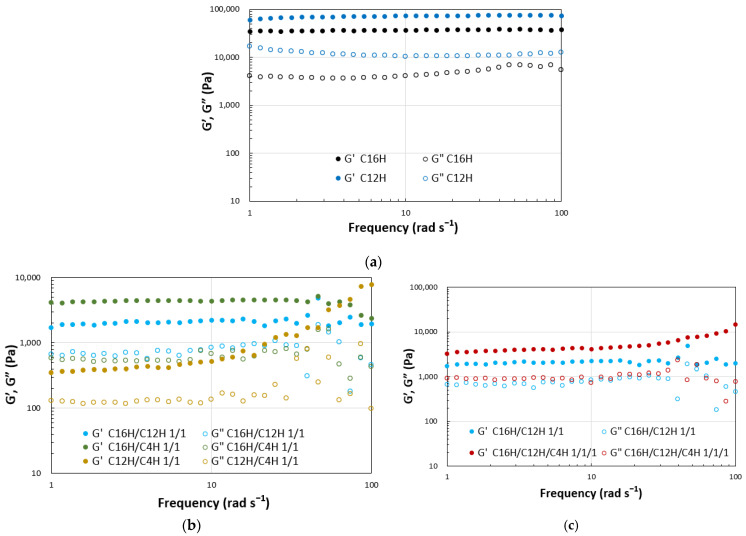
Dynamic rheological properties of the mixed molecular squalane organogels (3.0 wt.%) on frequency sweep; (**a**) single gels, (**b**) binary gels, and (**c**) tertiary gels with C16H/C12H 1/1 gel as a reference.

Dynamic viscoelasticity measurements were performed on the resulting gels to obtain a more detailed quantitative view of the abovementioned results. The results of strain dispersion measurements indicate that increasing the strain causes a clear transition from the gel state (G′ > G″) to the sol state (G′ < G″) in the two- and three-component systems, as demonstrated in general molecular gels [48]. The results reveal that the elastic modulus decreases and softens with mixing in the two- and three-component molecular gels. The strain increases during the gel-to-sol transition depending on the combination of components. These results indicate a match–mismatch combination of CnH mixing in the gel strength (Figure 3). Rheometry of the single and mixed molecular gels reveals that G′ > G″ over a wide range of frequency variation, confirming that these are in a gel state [49] in the same range of softness (Figure 4).

**Figure 5:**
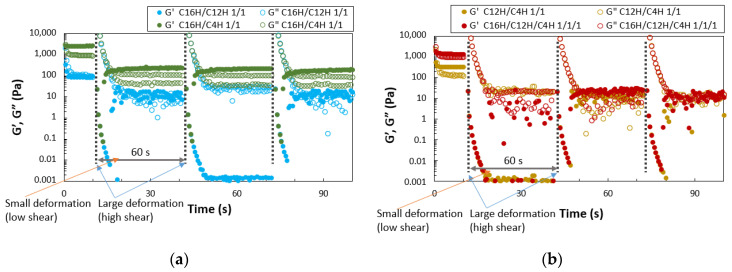
Periodic step-shear test results for the mixed molecular squalane organogels (3.0 wt.%); (**a**) C16H/C12H 1/1 gel and C16H/C4H 1/1 gel, (**b**) C12H/C4H 1/1 gel and C16H/C12H/C4H 1/1/1 gel.

The quantitative evaluation of thixotropy was also performed with rheometry by applying high and low shear (Figure 5). The results show a trend of repeated recovery from the sol state (G′ < G″) to the gel state (G′ > G″). However, a sharp decrease in the elastic modulus is observed after the initial application of external force. Compared with the two-component system, the three-component system has a more significant decrease in elastic modulus, and the plot values are less stable. Only the 16HDT/4HDT gel shows a clear, repeated recovery, indicating that it is a gel with good thixotropic properties (two plotted values of G″ at the same time can be attributed to the vibration of the gel). The C16H/C12H and tertiary systems show lower recovery than single gel systems, while binary mixed molecular gel systems show better recovery of gel state with larger G′ > G″ than single systems, especially in 16HDT/4HDT. Thixotropic behavior is observed in both the two- and three-component molecular gels in the vial test evaluation, but the step-shear test results do not show a clear recovery behavior from the sol state to the gel state. However, the rheometer test examined thixotropic behavior in a small space between flat plates, which does not correspond exactly to the vial test, but shows a tendency for G′ > G″ after large deformation in each system. C16H/C4H gel only shows stable behavior.

**Figure 6:**
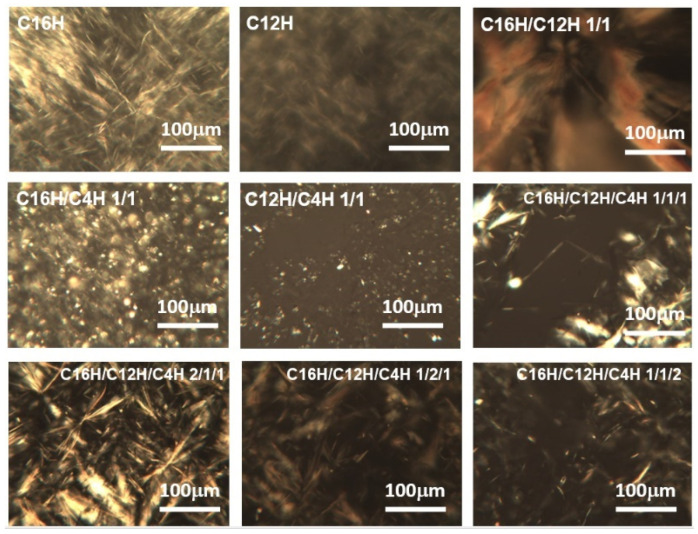
POM photos of the single and mixed molecular gels (solvent: squalane) under the crossed nicols.

**Figure 7:**
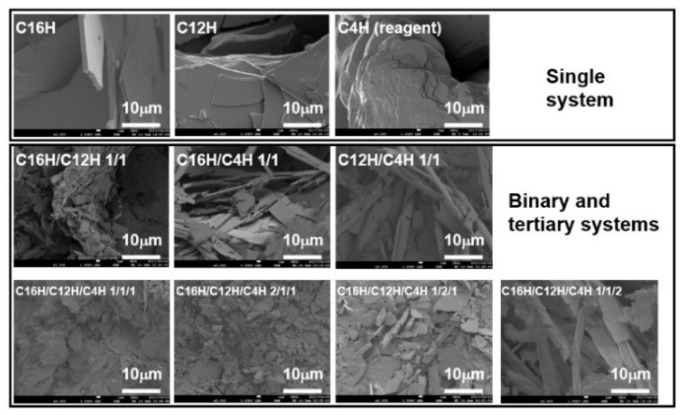
SEM images of the single and mixed xerogels obtained from molecular gels with *n*-octane as the solvent.

To investigate the inside structures of organogels, polarized optical microscopy (POM) and scanning electron microscopy (SEM) were conducted. From the results, gels consist of a network of crystalline fiber with tens of micrometers in width and 100 μm in length (Figure 6). Although these network structures and morphologies are observed in other kinds of gel systems [12], the mixed systems have a finer network than the single system, as seen in SEM images (Figure 7), which show a tape-shaped fiber with micrometer width in mixed systems, and a sheet-shaped fiber with dozens of micrometer width in a single system, a similar tendency shown in mixed molecular gel systems [28]. This change in the components of the gel mesh structure, i.e., the miniaturization of the components, may result in the densification of the mesh structure and, thus, the development of thixotropic properties. However, while there are examples of gelator fibers being changed by the introduction of a second component (surfactant) [50], it is still unclear how the structure of the constituent elements was changed by mixing.

**Figure 8:**
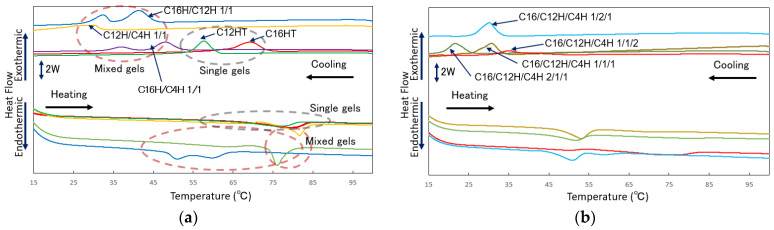
DSC curves of the mixed *N*-alkylhydantoin organogels. All samples are 3 wt.% squalane gels and mixed in a molar ratio (heating and cooling rate is 10 °C/min): (**a**) single and binary systems, (**b**) tertiary systems.

To further obtain information on the internal network structure of the molecular gels in the single and mixed systems, the thermal change behavior of the network structure was investigated by performing thermal analysis of the molecular gels by DSC measurements. In this thermal analysis, the gel sample dissolves to a sol state during the heating process and forms a gel state during cooling process. From the thermal analysis of the molecular gels in Figure 8 and Table 3, (1) an endothermic peak corresponding to the transition from gel to sol is observed during the temperature increase process of each sample, and an exothermic peak corresponding to the transition from sol to gel is observed during the high-temperature process, and (2) a temperature decrease in the peak of each transition is observed in the mixed gel compared to the single gel. Result (1) and corresponding **Δ**H values indicate that these transitions are reversible because the thermal energies (**Δ**H) of those processes are comparable. The DSC results also show that the three-component system has multiple to single exothermic and endothermic peaks in lower temperature regions compared to the two-component system, suggesting that the mesh structure is more uniform depending on the number of components. Also, result (2) shows a change in the internal network structure of the gel, which is thought to be due to the change of the network fibers into finer components, as the gel becomes more thermally changeable when mixed, which is consistent with the SEM observations. However, thixotropy evaluation results do not necessarily indicate that the microscopic homogeneity of the gel obtained by thermal analysis is advantageous for thixotropy, as the three-component system is advantageous for recovery in the vial test, and the two-component system results (C16H/C4H) are more stable plots in the rheometer test. To apply this squalane thixotropic gel to ointment base materials for health care, further study of the correlation between the internal structure of the gel and thixotropic properties is required.

**Figure 9:**
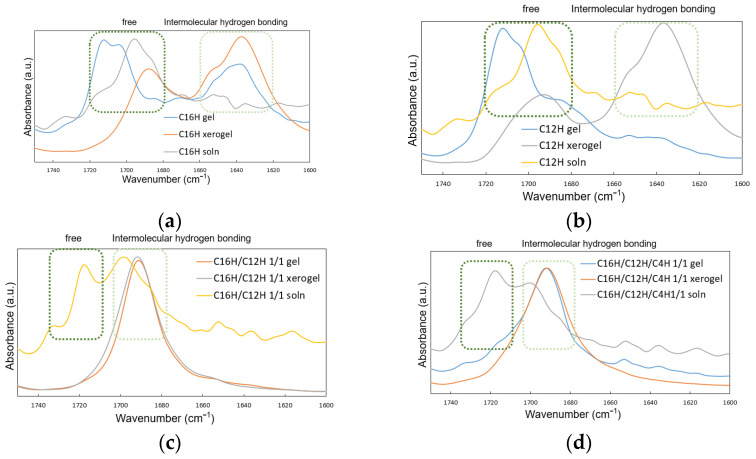
ATR–FTIR spectra of the *N*-alkylhydantoins in different states (a gel, aqueous solution, and xerogel) in the region of carbonyl stretching region: (**a**) single system C16H, (**b**) single system C12H, (**c**) binary system C16H/C12H 1/1, (**d**) tertiary system C16H/C12H/C4H 1/1/1.

Finally, by comparing the attenuated total reflectance Fourier-transform infrared (ATR–FTIR) spectroscopy absorption spectra in the gel, solution, and xerogel states among the gelators, the author observes how the hydrogen bonds function in each state. Figure 9 shows the ATR–FTIR spectrum of the absorption region of the carbonyl group involved in hydrogen bonding. In the single system (Figure 9a,b), the intermolecular hydrogen-bond-derived absorption peak in the xerogel decreases (near 1620~1660 cm^−1^), whereas the absorption peak of the hydrogen-bond-free species increases in the longer wavelength region in the gel and solution states (near 1680~1720 cm^−1^). In contrast, in the two-component and three-component gels (Figure 9c,d), only the hydrogen-bonding-derived absorption bands observed in the single system are observed in the xerogel and gel states (near 1710~1730 cm^−1^). Simultaneously, hydrogen-bonding and hydrogen-bond-free-derived absorption bands are observed in the solution state (near 1670~1710 cm^−1^ and 1710~1730 cm^−1^, respectively), indicating that the fiber formation by hydrogen bonding may be more robust in the mixed system than in the single system. This could be due to the difference in thixotropic behavior between the single system (without thixotropy) and the mixed system (with thixotropy), or the ease of fiber recovery from the sol state to the gel state after the gel network is broken by external mechanical force. The results and trends obtained in this study are observed in other mixed-system molecular gels [33,34,35].

## 3. Conclusions

In conclusion, the author demonstrates the gel properties of *N*-alkylhydantoins as LMWGs for molecular organogels for the first time, and the mixing strategy to enhance the gel-forming and mechanical properties compared to single gels. It is shown that *N*-alkylhydantoins become new LMWGs that exhibit the ability to gel hydrophobic organic solvents. Furthermore, improved thixotropic behavior is observed in mixed organogel systems, particularly in mechanical properties, even though each single molecular organogel exhibits poor thixotropic behavior, as evidenced by the rheometric results. The results in this study enable the commercial additives, *N*-alkylhydantoins, to be a new set of mixed molecular organogels and candidates for base materials of drug-release ointments. These new mixed molecular organogels with thixotropic behavior may be applied to ointment base materials in the health care and cosmetic fields, since they are obtained by gelling squalane oil, which is used as a base material for health care and cosmetic products. However, further research into the correlation between the homogeneity of the gel interior and thixotropic properties is required for future applications.

## 4. Materials and Methods

*N*-alkylhydantoins, 1-*N*-butylhydantoin (98%), 1-*N*-dodecylhydantoin (98%), and 1-*N*-hexadecylhydantoin (98%) were purchased from Tokyo Chemical Industry Co., Ltd., Tokyo, Japan. All other chemicals were purchased from Wako Pure Chemical Industries, Ltd., Tokyo, Japan, and used without further purification.

Gelation of *N*-alkylhydantoins was performed as follows. *N*-alkylhydantoins crystals were placed in a vial with a set concentration (wt.%) of solvent and capped. The vial was heated in a dry bath at 100 °C to dissolve the *N*-alkylhydantoins crystals. The solution was allowed to dissolve. The *N*-alkylhydantoins solution was allowed to stand at room temperature for 30 min to check for gelation. The mixed molecular gels of *N*-alkylhydantoins were tested by mixing *N*-alkylhydantoins homologues in integer molar ratios, as described in the main text.

Gelation ability and thixotropic behavior were evaluated by the vial inversion method (a method in which a vial containing gel is inverted and judged to be in a gel state if it does not drip). Thixotropic behavior is evaluated through examining if it recovered from a sol to a gel when it was broken with a vortex gene to form a sol and inverted after standing for a certain time (if it is not recovered and sol, it will drip).

POM observations of the organogels were carried out with a Leica DM2500 (Leica Microsystems GmbH, Wetzlar, Germany) polarized optical microscope under crossed nicols.

SEM image measurements were carried out using a SU-8000 scanning electron microscope (Hitachi High-Technologies Corporation, Tokyo, Japan) at 1.0 kV. The vacuum-dried organogels with octane as solvent (xerogels) for one day at 25 °C and then one day at 80 °C were measured on a conductive tape on the brass SEM stage. In addition, the samples were coated with a spattering of Pt (10 nm-thick) to add electrical conductivity.

Rheological measurements were performed using an MCR-301 rheometer (Anton Paar Japan K.K., Tokyo, Japan) with a parallel plate (8 mm diameter) at a gap of 0.50 mm at 25 °C. The frequency sweeps were carried out with γ of 0.01 %. The strain sweeps were carried out with a constant angular frequency of 1 rad s^−1^. The repeated step-shear measurements were carried out with a normal strain with an amplitude of 0.01% and frequency of 1 Hz, and a large strain with a shear rate of 3000 s^−1^ for 0.1 s.

Thermal analysis of organogels was carried out with an EXSTAR6220 differential scanning calorimeter DSC (Hitachi High-Tech Corporation, Tokyo, Japan) using an Ag-made closable sample pan.

ATR–FTIR spectra were recorded with a Cary 670 FTIR (Agilent Technologies Japan, Ltd., Tokyo, Japan) using a single bounce diamond ATR accessory.

## Figures and Tables

**Table 1:** Critical gel concentrations (CGC, wt.%) of *N*-alkylhydantoins in organic solvents.

Solvent	C16H	C12H	C4H
Propylene carbonate	K*^,^^1^	PG ^4^	S
*N*,*N*-Dimethyl formamide	K ^2^	S	S
Methanol	K	S	S
Ethanol	K	S	S
1-Butanol	K	S	S
Dichloroethane	K	S	S
Tetrahydrofuran	S ^3^	S	S
Ethyl acetate	K	S	S
Toluene	K	S	S
*n*-Octane	2	6	PG
Olive oil	3	5	5
Squalane	1	2	S

^1^ K*: crystallization at 5 wt.%, ^2^ K: crystallization at 10 wt.%, ^3^ S: solution at 10 wt.%, ^4^ PG: partial gel at 10 wt.%.

**Table 2:** Critical gel concentrations (CGC, wt.%) of mixed *N*-alkylhydantoin systems in squalane.

Mixed Molar Ratio	C16H/C12H	C16H/C4H	C12H/C4H	C16H/C12H/C4H
5/1	3	S ^1^	S	
2/1	2	2	S	
1/1	2	1	3	
1/2	2	1	1	
1/5	2	1	1	
5/1/1				3
1/5/1				2
1/1/5				1
2/1/1				3
1/2/1				3
1/1/2				1
1/1/1				2

^1^ S: solution at 3 wt.%.

**Table 3:** Transition temperatures of mixed *N*-alkylhydantoin organogels obtained by DSC measurements (heating and cooling rate is 10 °C/min).

Samples ^1^	T_gel to sol_ on Heating/°C (ΔH/mJ mg^−1^)	T_sol to gel_ on Cooling/°C (ΔH/mJ mg^−1^)
C16H	69.5 (6.4)	74.2 (6.4)
C12H	65.1 (5.0)	60.8 (5.0)
C16H/12H 1/1	47.1 (4.5) pt ^2^: 50.5, 59.7	45.6 (4.4) pt: 32.8, 41.7
C16H/4H 1/1	58.2 (5.8) pt: 66.8, 81.5	51.5 (5.1) pt: 36.8, 47.7
C12H/4H 1/1	56.9 (0.9) 73.7 (2.9)	31.7 (2.0)
C16H/12H/4H 1/1/1	47.0 (2.3)	30.5 (2.8)
C16H/12H/4H 2/1/1	44.2 (3.1)	25.7 (3.2)
C16H/12H/4H 1/2/1	43.9 (2.6)	33.4 (2.6)
C16H/12H/4H 1/1/2	45.3, 61.8 (3.6)	39.4 (3.3)

^1^ All samples are 3 wt.% squalane gels and mixed in molar ratio. ^2^ pt: temperature at peak top. Transition temperatures are defined as the onset temperature of the peaks of DSC curves.

## Data Availability

Not applicable.
